# Broadly Sourced Alternative Proteins Alter Muscle Metabolome While Maintaining Sensory Quality in Rainbow Trout (*Oncorhynchus mykiss*)

**DOI:** 10.1021/acs.jafc.5c07909

**Published:** 2025-10-22

**Authors:** Pontus Gunnarsson, Hanna Eriksson Röhnisch, Mihaela Mihnea, Aleksandar Vidakovic, Markus Langeland, Anders Kiessling, Johan Dicksved

**Affiliations:** † Department of Applied Animal Science and Welfare, 8095 Swedish University of Agricultural Sciences, 756 51 Uppsala, Sweden; ‡ Department of Molecular Sciences, Swedish University of Agricultural Sciences, 750 07 Uppsala, Sweden; § Sense Lab, School of Hospitality, Culinary Arts and Meal Science, Örebro University, 701 12 Örebro, Sweden; ∥ Department of Food Research and Innovation, 388792 RISE Research Institutes of Sweden, 402 29 Göteborg, Sweden

**Keywords:** metabolomics, sensory, Rate-All-That-Apply, RATA, rainbow trout, *Oncorhynchus mykiss*, alternative feeds, fillet, 1H NMR

## Abstract

Aquaculture is recognized as a key future food source, and to sustain increasing production and reduce the ecological footprint, new protein sources are needed. However, replacing the conventional fish and soybean meals may affect fillet quality. This study examined a phylogenetically broad set of protein sources and their effects on sensory attributes and metabolism of rainbow trout (*Oncorhynchus mykiss*). Five feeds derived from microfungi, black soldier fly, mealworm, blue mussel, and tunicate were evaluated against a soy-based control. Sensory attributes were assessed using Rate-All-That-Apply, and ^1^H NMR metabolomics to analyze feed and muscle metabolomes. Phylogenetically closer protein sources showed more similar feed and muscle metabolite profiles, and only minor differences in color, root vegetable taste, and aroma were found. Metabolic effects involved osmoregulation, energy, and amino acid metabolism. Overall, insect-based feeds appeared most favorable based on sensory results and product quality biomarkers; however, all tested feeds remained viable.

## Graphical abstract

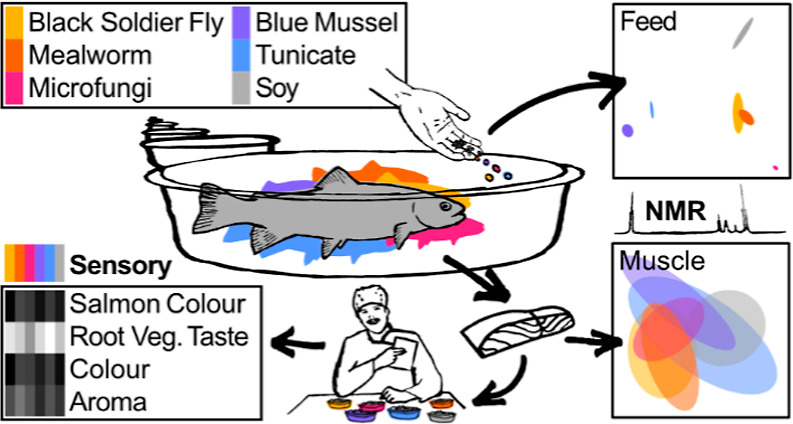

## Introduction

Aquaculture in the global food market has never been as relevant as it is today. Capture fisheries have stagnated in landed volumes, and in 2022, aquaculture production surpassed capture fisheries for the first time,^1^ a historic event highlighting aquaculture’s role in providing a growing world population with a nutritious diet. On top of this, cropland is becoming a less available resource^2^ while global meat consumption is increasing.^3^ These trends have raised concerns for the future environment and public health^4^ and call for a shift in consumption to other food sources. The list of factors paving the way for increased aquaculture is long, but there are also challenges. A key issue in the ecological footprint of finfish aquaculture is feed. Today, fish feed is partly produced from raw materials competing with our direct consumption and land use.^5,6^ The risk of overfishing stocks for fish meal endangers natural populations,^7^ and soybean, which is a common imported protein source in feeds in the EU,^8^ is strongly associated with rainforest deforestation and CO_2_ emissions.^9,10^ In a Scandinavian context, available salmon feed consists of a smaller share of marine sources (about 22%) and a larger share of vegetable-based materials (∼73%).^11^ The future of aquaculture is highly affected by the availability of raw material, given that the goal is a lower ecological footprint.^12,13^

Typically, alternative proteins serve as substitutes for soybean or fish-derived ingredients, aiming to reduce ecological impact, and the candidates represent a broad spectrum of phylogenetic origins.^14–17^ Relevant groups include insects, crops, fungi, and marine filter feeders with unique strengths and weaknesses. Some microfungi, such as *Paecilomyces variotii*, show strong potential as protein converters of various substrates, including forestry side-streams, highly relevant to a Scandinavian setting.^18^ From a protein source perspective, microfungi share several characteristics with insects such as black soldier fly (*Hermetia illucens*) and mealworm (*Tenebrio molitor*). In addition to their substrate flexibility, both insects and microfungi are characterized by scalable, season-independent production and rapid growth.^19,20^ A challenge common to microfungi and insects is the presence of rigid structural components, particularly chitin, which may reduce digestibility if not adequately processed.^21,22^ Similarities aside, black soldier fly and mealworm have higher fat contents than *P. variotii*, which can be advantageous for fish species with high lipid requirements, while it also may pose practical challenges during extrusion.^23^

Several species of marine suspension feeders, such as the blue mussel (*Mytilus edulis*) and tunicates like the sea vase (*Ciona intestinalis*), are commonly found along the Swedish west coast, with the blue mussel also reaching into the brackish southern Swedish east coast. Both species are slow growers in comparison to insects and microfungi,^24,25^ but they play a valuable role in nutrient uptake in the often eutrophic coastal waters.^26^ While tunicate farming is a recent development, the use of farmed west coast blue mussels for human consumption is vast, where damaged mussels, or those that fall outside the acceptable size range, are considered nonmarketable. Moreover, due to the high energy demands of osmoregulation for the brackish-farmed east coast populations, the growth stagnates at sizes less suitable for human consumption.^27^ In both the east and west coast cases, the production will generate mussels that are more suitable for fish feed than for our plates. In contrast to insects, blue mussels and tunicates contain relatively little fat,^28,29^ which is advantageous when producing extruded feeds. Despite the low lipid content, the existing fat fraction is rich in valuable *n*-3 polyunsaturated fatty acids.^30,31^ Instead of chitin found in insects and microfungi, the tough structural components in blue mussels are the shells, contributing to an increased ash content of the feeds,^28^ while for tunicates, it is the cellulose content that reduces the nutrient density.^32^ While the conventional proteins used in salmonid feed are cheaper today, the exemplified protein sources provide alternatives that do not compete with our consumption. Rather, they play a key role in turning side-streams and excess nutrients into high-quality food items.^33^

Sensory quality is a critical determinant of consumer acceptance, and in the context of fish farming, feed choice is one way to influence sensory attributes of the final product.^34^ Here, texture and flavor are particularly interesting for the producers as they may directly affect marketability.^35^ One increasingly employed sensory method to assess product attributes in a detailed and structured way is Check-All-That-Apply (CATA), in which panellists indicate the presence or absence of predefined, product-relevant attributes.^36^ The more advanced version of CATA is Rate-All-That-Apply (RATA), which replaces binary responses (“detected” or “not detected”) with intensity ratings, resulting in a more detailed product description.^37^ These methods can provide valuable insights into how novel feed formulations influence the end-product, allowing for better predictions of consumer acceptance. Understanding sensory impacts is of high importance, as consumer willingness to purchase fish fed alternative proteins is often tied to perceived taste and texture.^38^ Although clear communication of sustainability benefits can improve consumer acceptance, even when attributes such as meat color are affected,^39^ the best solution is to balance maintaining product characteristics while reducing ecological impact and ensuring a healthy diet.

Beyond sensory perception, the metabolite composition of meat products plays a crucial role in defining their sensory attributes. In fish, metabolites such as carbonyls and alcohols are linked to fresh flavors,^40^ volatile sulfur compounds to both pleasant and undesirable odors,^41^ and the typical “fishy smell” originates from the bacterial-driven accumulation of trimethylamine (TMA) as muscle tissue deteriorates.^42^ Sensory attributes are, therefore, partially reflective of a product’s unique metabolite profile.^43^

In this study, we aim to compare the sensory attributes, muscle metabolomes, and the links between these factors in rainbow trout fed a diverse range of alternative protein sources. The proteins used are expected to produce feeds with different metabolite profiles, which we hypothesize can influence the muscle metabolome and, consequently, the fillet’s sensory attributes. By integrating sensory evaluation methods like RATA with metabolomic profiling, we seek to clarify how these alternative feeds impact not only the sensory quality of the final product but also its underlying chemical composition. Such insights are critical for optimizing novel feed formulations to meet consumer expectations of the fillets.

## Material and Methods

### Feed Production

One commercial-like control and five experimental diets were formulated for this trial (Table 1). Each experimental diet contained one of the five test ingredients (blue mussel meal (*M. edulis*; MM), tunicate meal (*C. intestinalis*; CIO), mealworm meal (*T. molitor*; MW), black soldier fly meal (*H. illucens*; BSF), and a single cell protein (the filamentous microfungi *P. variotii*; MF) replacing soy protein concentrate on a crude protein basis. The exception was diet CIO, where the soy protein concentrate has been replaced by a mixture of mussel meal and tunicate meal in order to minimize the chances for palatability issues that may be caused by tunicate meal, as was observed by Warwas et al. (In preparation). All test feeds were formulated to be similar in their protein, lipid, and energy contents (Table 2), and to fulfill the nutrient requirements of rainbow trout.^44^

**Table 1:** Feed Formulation (on As-is Basis) of the Control- and Five Experimental Feeds Produced

ingredient	BSF	MW	MF	MM	CIO	CTRL
fish meal (g/100 g)	20.00	20.00	20.00	20.00	20.00	20.00
wheat gluten (g/100 g)	15.00	15.00	15.50	15.50	16.00	15.50
wheat meal (g/100 g)	14.00	14.00	14.00	14.00	13.50	14.00
potato starch (g/100 g)	5.50	7.40	0.60	1.50	0.00	1.50
fish oil (g/100 g)	14.00	14.00	14.00	14.00	14.00	14.00
rapeseed oil (g/100 g)	3.00	3.50	4.00	4.00	4.50	5.00
black soldier fly meal (g/100 g)	25.60					
mealworm meal (g/100 g)		23.20				
P. variotii meal (g/100 g)			29.00			
mussel meal (g/100 g)				28.30	15.10	
ciona meal (g/100 g)					14.00	
soy protein concentrate (g/100 g)						27.30
vitamin mineral premix (g/100 g)	0.90	0.90	0.90	0.90	0.90	0.90
dl-methionine (g/100 g)	0.70	0.70	0.70	0.55	0.70	0.55
monocalcium phosphate (g/100 g)	1.00	1.00	1.00	1.00	1.00	1.00
choline chloride (g/100 g)	0.30	0.30	0.30	0.30	0.30	0.30

**Table 2:** Proximate Composition and Energy Content of the Control- and Experimental Feeds on an As-is Basis

proximate composition	BSF	MW	MF	MM	CIO	CTRL
dry matter (%)	94.5	93.7	93.9	93.1	94.0	94.7
crude protein (% dry matter)	43.7	42.5	42.1	44.5	41.1	44.3
crude fat (% dry matter)	18.4	19.2	19.8	18.0	18.1	19.1
neutral detergent fiber (% dry matter)	2.90	2.20	1.10	1.10	1.70	1.30
ash (% dry matter)	5.90	5.00	6.30	9.60	12.0	6.10
gross energy (MJ/kg DM)	23.4	23.6	23.2	21.9	21.1	23.0

All feeds were produced by extrusion on a twin-screw extruder (4 mm die, Ketse 20/40, Brabender GmbH & Co. KG, Duisburg, Germany) followed by the addition of oil by vacuum coating (Mini-GVC 10, Amandus Khal, Reinbek, Germany) at the Feed Technology Laboratory, Department of Applied Animal Science and Welfare, Swedish University of Agricultural Sciences, Uppsala, Sweden.

### Experimental Design

The experiment was conducted at Vattenbrukscentrum Norr AB (in Kälarne, Sweden) between September 2021 and February 2022 using 360 rainbow trout (start weight 556.8 ± 71.2 g; mean ± SD). The fish were hatched in-house, reared, and marked by PIT-tag (Passive Integrated Transponder; Biomark, Boise, Idaho, USA) under mild anesthesia (40 mg/L) with tricaine methane-sulfonate (MS-222; Western Chemical Inc., Ferdale, WA, USA). The fish were given commercial feed in a common holding tank, and for 2 weeks in the experimental tanks, before the experiment started. At the start, the fish were anesthetized (MS-222; 40 mg/L), weighed, and randomly allocated to the experiment tanks. A total of 24 square (98.5 × 98.5 × 64 cm) 340 L tanks (*n* = four tanks per diet) were used, housing 15 individuals in each. Water was supplied from Lake Ansjön, passing a drum filter and heater at a system flow rate of 10 L/min and a daily measured temperature of 11.6 ± 2.7 °C (mean ± SD). The six feeds were randomly assigned to four tanks per feed, and the fish were fed to satiation daily for 18 weeks using automatic feeders with manually set rations, under a 12:12 light cycle. The experiment, and associated animal husbandry practices, were approved by the Ethics Committee for Animal Experiments in Uppsala (Uppsala djurförsöksetiska nämnd; Approval No. 5.8.18-23275/2022) and by the laws and regulations overseen by the Swedish Board of Agriculture.

### Sampling and Sensory Evaluation

For the final sampling, eight fish were randomly selected from two tanks (four per tank) from each treatment (*n* = 48). Euthanasia was performed by percussive stunning followed by gill arch exsanguination, after which each fish’s body weight was recorded (final weight 1139.0 ± 251.9 g; mean ± SD for all fish). The fish was gutted, rinsed, and stored on ice until sensory evaluation. Four samples of each feed (*n* = 24) were also collected for metabolomics analysis.

The cleaned fish was transported on ice to Campus Grythyttan, School of Hospitality, Culinary Arts and Meal Science, Örebro University. On the day of sensory evaluation, all 48 fish were filleted and diced (*Macédoine* cut). The diced meat from each fish was divided into several small white plastic bowls, each containing roughly two tablespoons of meat from one fish only. The bowls were then sealed with plastic wrap and stored refrigerated at 4 °C. Simultaneously, a one-centimeter-thick Norwegian quality cut quarter was cut out from the posterior end of the dorsal fin (Figure 1), from which the center (white skeletal muscle tissue) was stamped out using an 8 mm biopsy punch (Kai Europe GmbH, Solingen, Germany). Muscle samples were frozen in liquid nitrogen immediately after collection, transported to the laboratory, and stored at −80 °C until analysis.

**Figure 1:**
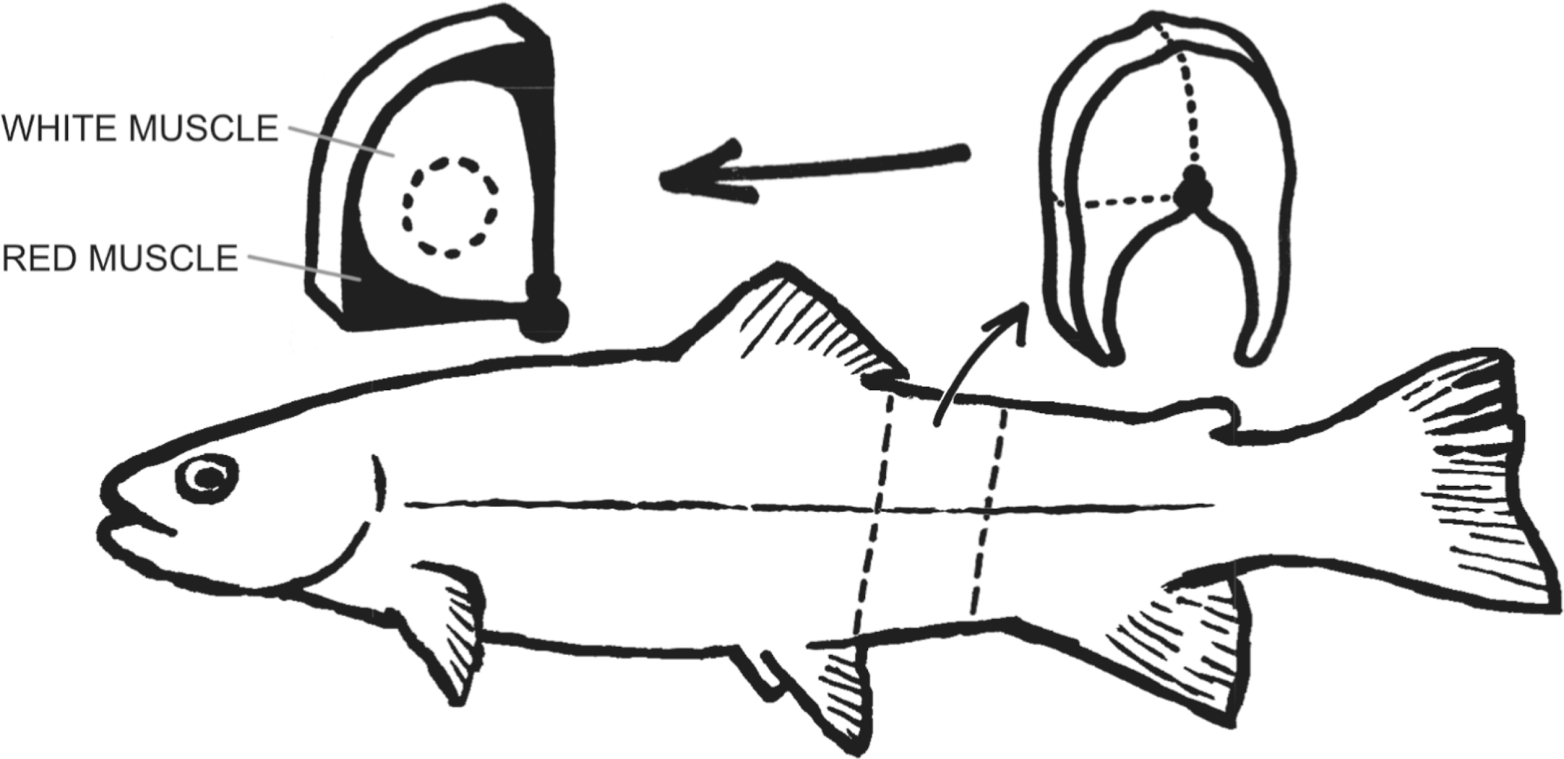
Sampled area for white skeletal muscle tissue. The dotted lines indicate the biopsy punch location.

Around half an hour before serving, the fish samples were brought to room temperature. Trained assessors with prior experience in sensory evaluation were invited through an open call distributed by Örebro University, and all participation was voluntary. All assessors (*n* = 27) were served 12 white plastic bowls of tartar (six diets × two replicates), each labeled with a unique three-digit code. In total, each fish was assessed by four to eight judges. The time between slaughter and sensory assessment was approximately 50 h.

The attributes used for sensory profiling were taken from an earlier assessment of rainbow trout created by sensory experts at Örebro University. The complete list consisted of 24 attributes, left undefined and open to interpretation, spanning four categories: taste (12 attributes), aroma (9 attributes), appearance (2 attributes), and texture (1 attribute). The attribute intensity was reported as a rate-all-that-apply (RATA) score using a 5-point scale (1 = very low, 3 = moderate, 5 = very high) with the option of a 0-score if the attribute could not be detected. The scores were collected using the EyeQuestion software (v. 5, Logic8 Elst, The Netherlands). Assessors were given a randomized sample order and were provided with water, crackers, and a one-minute break between products.

### NMR-Based Metabolomics

Sample preparation was done according to Brunel et al.,^45^ with slight protocol modifications. From each feed and muscle tissue sample, 200 mg of material was homogenized in 800 and 170 μL of ice-cold methanol and chloroform, respectively. The homogenization was performed using 2 mL Precellys lysing kits CK14 and a Precellys 24 tissue homogenizer (Bertin Technologies, Montigny-le-Bretonneux, France). The program was set to two cycles at 5500 rpm for 20 s with a 10 s rest between runs, followed by cooling on ice for 2 min, then repeated. After sonication in an ice bath for 30 min (Elmasonic S30H; Elma Schmidbauer GmbH, Germany), 800 μL ice-cold chloroform and 0.4 μL ice-cold water were added. The homogenate was then vortexed for 1 min and centrifuged for 10 min (2000*g* and 4 °C; HERMLE Larbortechnik Type Z 383 K, Germany). After separation, the polar phase was extracted, dried (Automatic Environmental Speed Vaac; Savant Instruments, Inc., USA.), and dissolved in 520 μL sodium phosphate buffer (0.135 mol/L, pH 7.22). The mixture was then filtered through a Nanosep 3K omega filter unit (Pall Life Sciences, New York, USA) using a centrifuge (Eppendorf 5424R, Eppendorf AG, Hamburg, Germany) in three intervals with cooling between (3 × 3 min at 12,000*g* and 20 °C). Finally, 50 μL D_2_O, 30 μL internal standard (trimethylsilyl-*d*_4_-propionic acid, TSP; 5.8 mmol/L), and buffer top-up of 260 μL were added to the filtrate, out of which 560 μL was transferred to NMR tubes.

The one-dimensional ^1^H NMR spectra for each muscle and feed sample were acquired using a Bruker Avance III 600 MHz spectrometer (Bruker, Karlsruhe, Germany) at 25 °C. The chosen pulse program was *zgesgp* with 128 scans spanning a spectral width of 9615 Hz with a 1.7 s acquisition time and 4.0 s relaxation delay. The data was Fourier-transformed in Bruker Topspin 3.5 before applying manual phase-, baseline-, and shim correction, followed by a 0.3 Hz line broadening and CSI calibration using ChenomX NMR Suite 9 processor (ChenomX Inc., Edmonton, Canada). The processed spectra were exported to the ChenomX profiler for compound identification and quantification with the Human Metabolome Database (www.hmdb.ca) and a set of articles as identification support.^46–48^ Quantification was done manually in ChenomX based on a previously described strategy.^49^

### Statistical Analysis

All data management and statistical analyses were performed using R (v. 4.3.3).^50^ From the metabolomics data sets, a total of 57 and 81 metabolites were identified in the muscle and feed samples, respectively. Metabolites that did not pass quality tests were removed (*n*_muscle_ = 8, *n*_feed_ = 21). These included metabolites that could yield unreliable quantification results because of sample preparation challenges or difficulties regarding detection and spectral fitting. For any samples of lower initial mass (<200 mg; 3 muscle samples), the concentrations were recalculated. For all samples, muscle metabolites with more than 20% missing values were removed, and remaining zero- or missing values were replaced with one-fifth of the lowest concentration within the compound. A total of 49 muscle and 60 feed metabolites passed quality control, whereof four muscle metabolites had >20% missing values. The data was then log10-transformed, mean-centered, and unit variance scaled. Outliers were identified using PCA-Hotelling *T* ^2^ (95% confidence interval),^51^ resulting in the removal of three observations of muscle tissue (*n* = 45) and none for feed (*n* = 24). A normal probability plot and a histogram of the PCA model were used to confirm normality after data filtering.

To determine diet-related differences between the metabolomes of muscle tissues and feeds, sparse partial least-squares discriminate analysis (sPLS-DA) from the mixOmics package was applied (R package version 6.30.0).^52^ Following normality assessment using Shapiro–Wilk tests, individual muscle metabolite concentration differences were analyzed using nested ANOVA, with tank nested within diet. For this analysis, metabolites with more than 20% missing values were included. Multiple testing was corrected using false discovery rate (FDR) *p*-value adjustments, and metabolites primarily associated with tank variation were excluded. Tukey’s honestly significant difference was applied to identify the pairwise differences for metabolites showing concentration differences driven by diet. The α-level was set to 0.05 for adjusted and unadjusted *p*-values for all tests.

A mixed effect model was estimated through maximum likelihood to identify sensory attribute intensity differences between diet groups and thus also extract attributes of higher flavor relevance. The models were created using lmer from the lme4 package^53^ and included sensory attributes as response variables, feed treatment as a fixed effect, and judge as a random effect. The reduced comparative models had feed treatment removed, and the full and reduced models were then compared using ANOVA with maximum likelihood model refitting. For the sensory attributes showing a significant impact of diet, estimated marginal means were calculated for the feed groups and pairwise compared under Tukey’s honestly significant difference adjustment (R package “emmeans”).^54^ Again, multivariate analysis was performed using sPLS-DA using averaged sensory scores over each fish for each measured attribute. The procedure of normalization, outlier detection, and statistical analysis followed the same method as above, with no outliers removed (n = 48).

Conditional inference trees (CIT) from “partykit” (R package version 1.2.23)^55^ were used to identify potentially important muscle metabolite predictors to the four sensory attributes that differed in intensity between diet groups. This decision tree model identifies patterns between multiple independent and one dependent variable. It does this by determining the optimal split for an independent variable and the specific value within that variable to create two groups that are considered homogeneous within themselves. Once a splitting point is found, the procedure is recursively applied to the two resulting subsets. The stepwise splitting keeps creating a branched structure until no statistically relevant data splits can be made at α-level 0.05.^56^ This statistical hypothesis testing inherent to CIT is a key reason for choosing this in favor of traditional decision tree methods. The fusion of the two data sets resulted in the listwise deletion of three observations (*n* = 45), and four metabolites were excluded based on a high abundance of missing values (>20%).

## Results

### Feed Metabolites

There were clear differences in metabolite profiles between the different feed samples, as shown by the distinct clustering in Figure 2. The exception is the similarity of insect feeds, where only slight variations can be seen in the concentrations of metabolites along components one and two (Figure 2). The differences between the insect feeds are most pronounced in component three, where MW stands out from the other feeds in having higher concentrations of, for example, betaine, adenosine monophosphate (AMP), and TMA.

**Figure 2:**
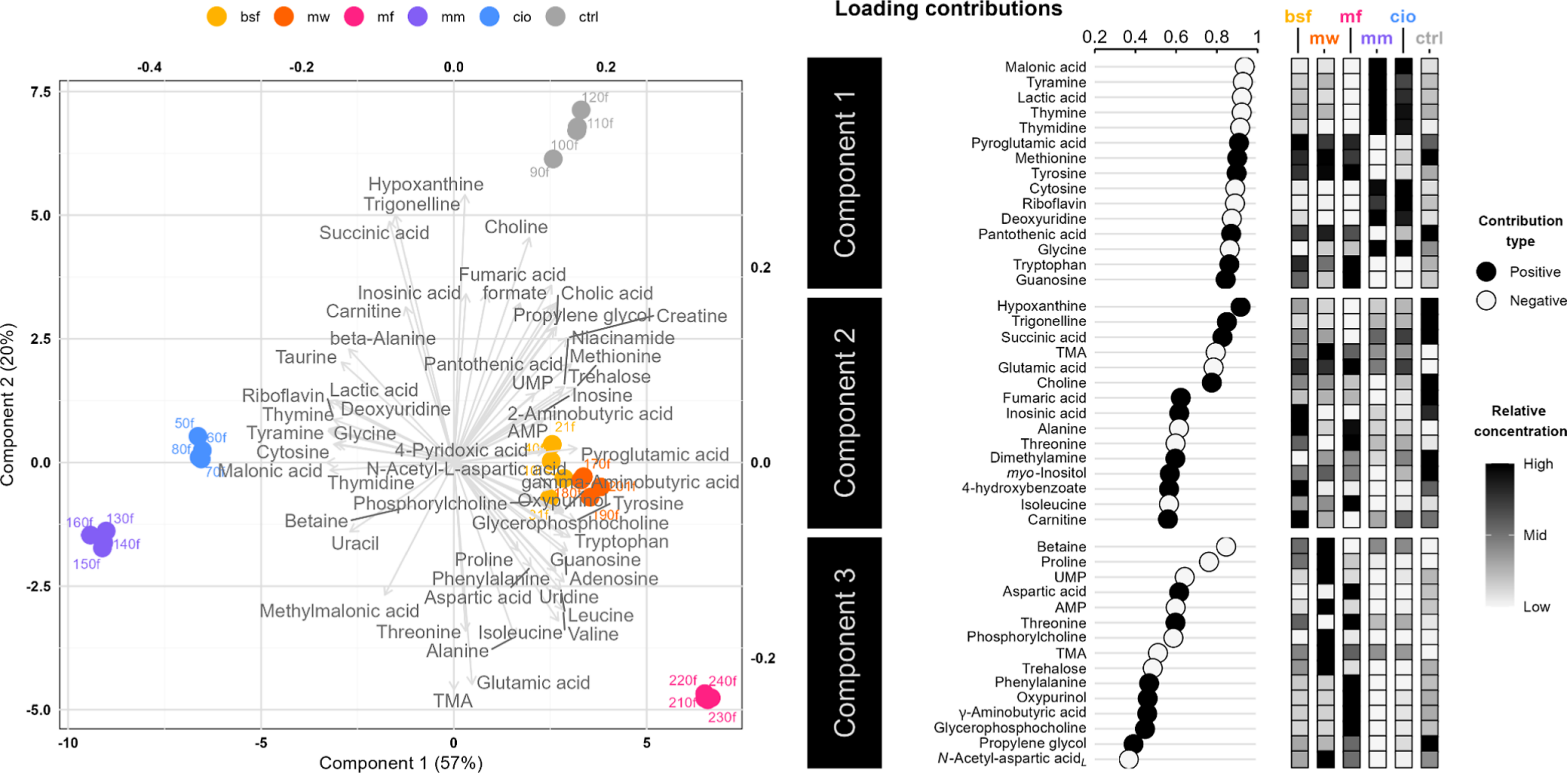
Sparse partial least-squares discriminate analysis (sPLS-DA) of metabolite profiles for the soy-based control feed and protein fraction replaced experimental variations. A biplot of metabolite loadings and observations is shown for components one and two, explaining 54 and 22% of the variance, respectively. The 15 metabolites with the strongest influence on components one to three are presented as positive or negative contributions, alongside their relative concentrations across the different feeds. BSF, black soldier fly; MW, mealworm; MF, microfungi (*P. variotii*); MM, blue mussel; CIO, tunicate; CTRL, control (soybean).

The two marine feeds (MM and CIO) differentiate from the others mainly along component one, driven by higher levels of metabolites such as tyramine, thymine, and lactic acid (Figure 2). Among these metabolites, MM generally contained higher levels than CIO. Additionally, both marine feeds shared a comparatively lower methionine, pyroglutamic acid, pantothenic acid, and creatine content than the other feeds. Again, MM differs slightly from CIO for these compounds, this time through consistently lower concentrations. Along component two, no clear patterns uniquely distinguish the marine feeds.

Along component two in Figure 2, CTRL and MF represent the extremes. Compared to the other feed groups, CTRL generally exhibits higher levels of, for example, hypoxanthine, trigonelline, and succinic acid, while showing lower levels of TMA and glutamic acid (Figure 2). In contrast, MF was characterized by higher levels of glutamic acid, alanine, isoleucine, and valine. Additionally, MF differed most evidently from most other feeds along component three (Figure 2).

### Muscle Metabolites

Forty-five compounds of the polar fraction of muscle extracts were quantified from ^1^H NMR spectra. One observation from the CIO diet group, and two from MW were detected as outliers by Hotelling’s *T* ^2^ and were removed. In the sPLS-DA analysis of the muscle metabolome (Figure 3), the first component accounted for 13% of the total variance, while the second component explained 12%. Like the feed metabolite profile, the two insect groups appear similar in composition of muscle metabolites, as the observations overlapped quite extensively. The most evident difference between metabolite profiles, however, could be seen between the control and the insect- and microfungi diet groups, with apparent separate clustering and no overlap, i.e. the insect- and microfungi-based feeds seem to have the most substantial influence on muscle metabolite profile in comparison to the control. The CIO group overlapped the control observations to the greatest extent. Still, both MM and CIO took on a similar spread, traversing the center in a similar pattern, differentiating slightly from both the insect treatment groups and the control in occupied space.

**Figure 3:**
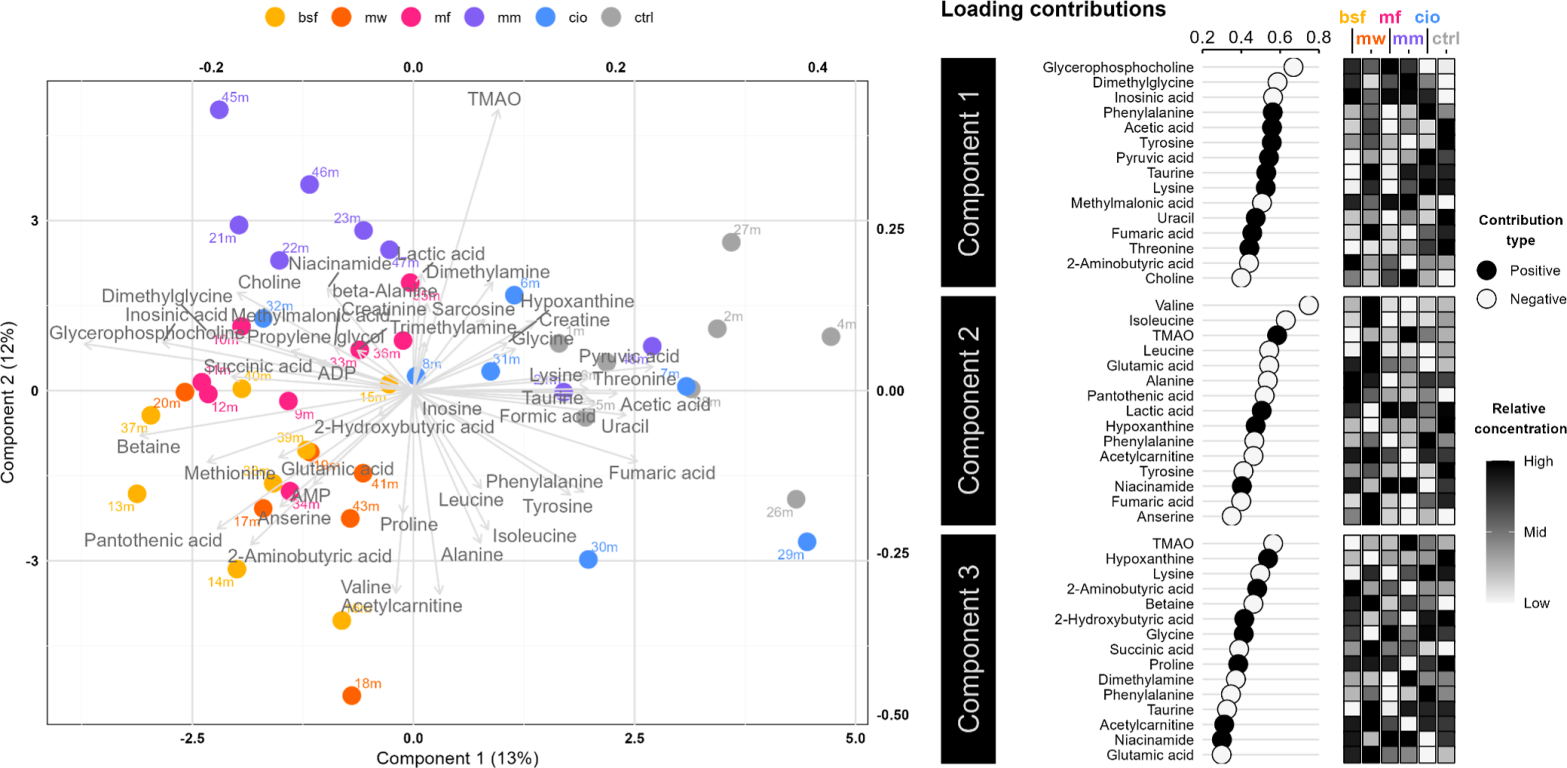
Sparse partial least-squares discriminate analysis (sPLS-DA) of muscle metabolome for rainbow trout fed a soy-based control diet or a protein fraction-replaced experimental diet. A biplot of metabolite loadings and observations is shown for components one and two, explaining 12 and 11% of the variance, respectively. Positive and negative loading contribution to components 1–3 is shown for the 15 most influential metabolites, together with the relative concentration of each metabolite per feed group. BSF, black soldier fly; MW, mealworm; MF, microfungi (*P. variotii*); MM, blue mussel; CIO, tunicate; CTRL, control (soybean).

A variance analysis was performed for each profiled compound, identifying six metabolites (betaine, hypoxanthine, malonic acid, methylmalonic acid, trimethylamine-*N*-oxide (TMAO), and 2-hydroxybutyric acid) showing significant differences between groups attributed to the diet (Figure 4). These metabolites were also among the highest contributing loadings to sPLS-DA components 1–3 (Figure 3).

**Figure 4:**
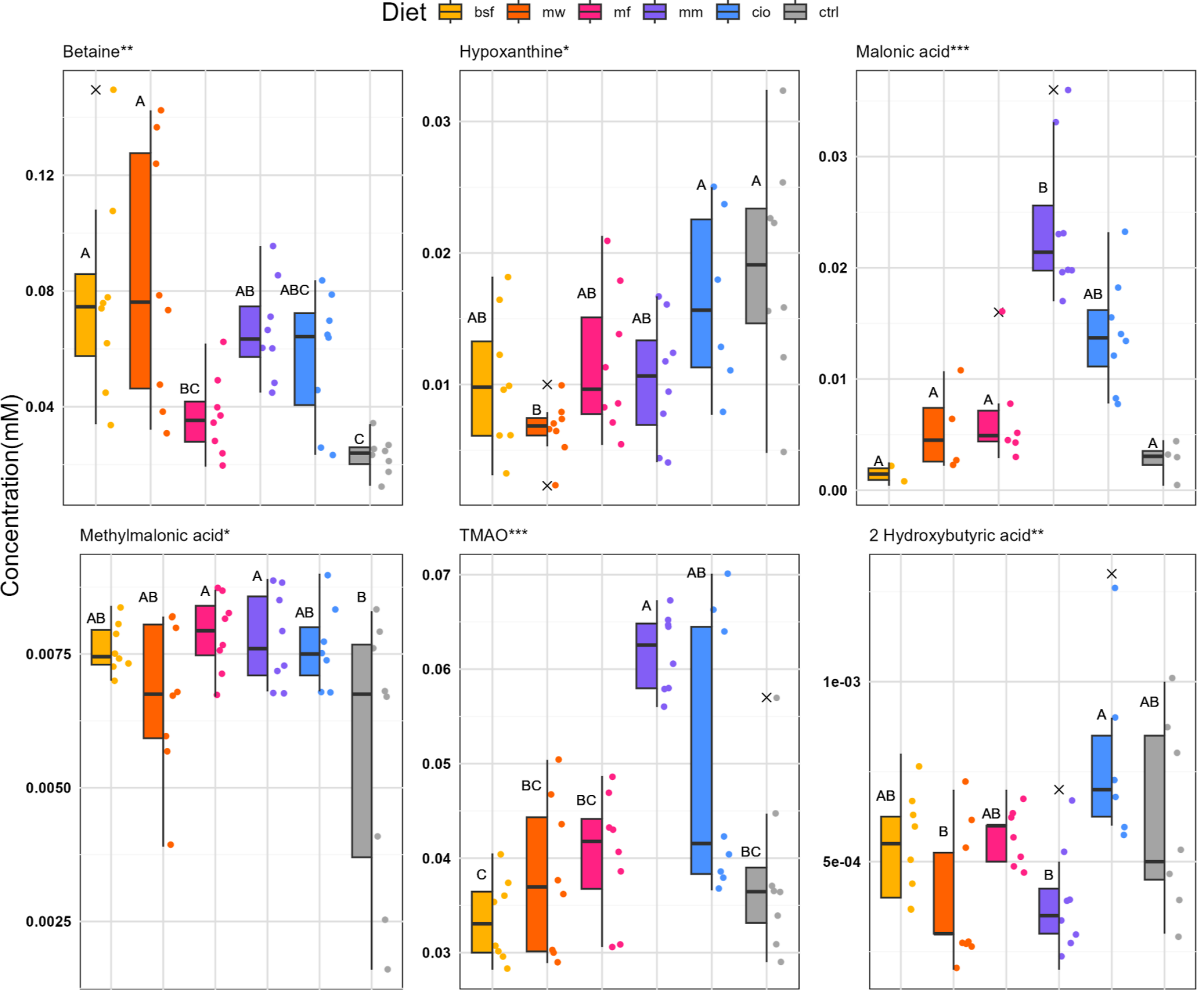
Relative concentrations of polar muscle tissue metabolites for significantly different diet groups, represented by a combined box-, whisker-, and jitter plot. Boxes represent the interquartile interval (50% of data) around the median. Error bars are extended to the most extreme value within the 1.5× interquartile range. Observations are shown as colored points, and those outside the range (outliers) are marked by a cross. Compact letters (a–c) not shared between groups indicate a significant difference. BSF, black soldier fly; MW, mealworm; MF, microfungi (*P. variotii*); MM, blue mussel; CIO, tunicate; CTRL, control (soybean). Asterisks represent *p*-values after FDR-adjustment as ***< 0.001, ** <0.01, *< 0.05.

### Sensory Assessment

The sPLS-DA analysis showed no evident clustering differences in RATA sensory profiles between the diet groups (Figure 5). The inclusion of insect-, microfungi-, and marine-based proteins resulted in minimal sensory deviations compared to the soy-based control. Attributes like fresh fish aroma, shellfish aroma, and oceanic aroma remained consistent across the dietary treatments, while slight increases in root vegetable aroma and taste were observed in samples from the microfungi diet, indicating a notable, yet mild, influence on flavor. These results suggest that the alternative proteins caused only minor deviations in sensory attributes, which are unlikely to influence overall acceptability.

**Figure 5:**
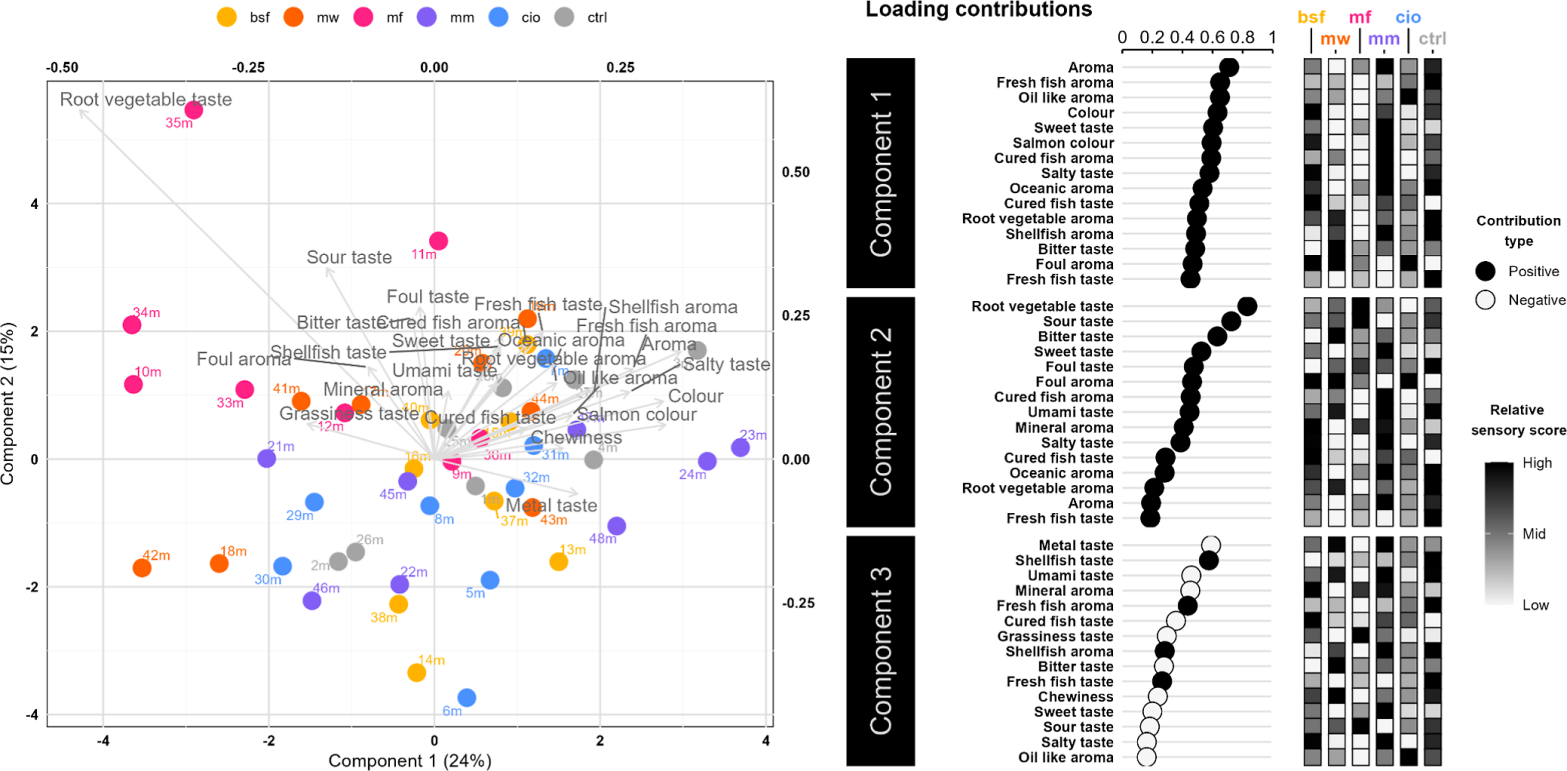
Sparse partial least-squares discriminate analysis (sPLS-DA) of rainbow trout meat sensory scores. Different colors represent different feed treatment groups, and the loadings are sensory attributes. The first two components are shown in this biplot, contributing 24 and 15% to the variance explanation. For components 1–3, the 15 most positively or negatively contributing sensory attributes are presented. The average fillet sensory scores are shown on a relative scale across all feed groups and for each attribute. BSF, black soldier fly; MW, mealworm; MF, microfungi (*P. variotii*); MM, blue mussel; CIO, tunicate; CTRL, control (soybean).

After comparing the mixed models explaining sensory attributes with and without diet as effects, four sensory attributes showed a statistical model improvement when including the diet variable. These were: color intensity (*p* < 0.001), salmon color intensity (*p* < 0.001), aroma intensity (*p* = 0.005), and root vegetable taste (*p* < 0.001; Appendix Table C1). Most estimated scores fell within the range of zero (attribute not detected) to three (moderately intense; Figure 6).

**Figure 6:**
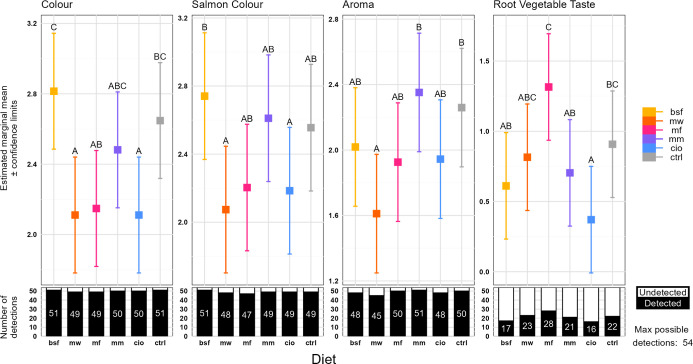
Sensory attribute scores for rainbow trout tartar. Within each sensory attribute, the estimated marginal means with confidence limits are shown for each diet group (BSF, black soldier fly; MW, mealworm; MF, microfungi (*P. variotii*); MM, blue mussel; CIO, tunicate; CTRL, control (soybean)). Letters not shared between the two groups indicate a difference between diets. The numbered lower bars give the number of times (out of 27 × 2 possible identifications) judges scored a nonzero intensity for each attribute and diet.

The differences found for overall color intensity and salmon color intensity were generally similar, with the BSF group showing higher intensity than the MW and CIO groups in both. In addition, BSF received a higher score than MF for overall color intensity, and the CTRL group showed a higher overall color intensity than MW and CIO. For overall aroma intensity, the MM and CTRL groups had a stronger aroma than the MW group. Root vegetable taste was more pronounced in MF compared to BSF, MM, and CIO, which is also valid for the CTRL compared to the CIO group.

### Associations between Metabolite and Sensory Data

A conditional inference tree (CIT) analysis was conducted to find metabolite patterns explaining the scores of the four sensory attributes. The most impactful metabolite explaining color intensity was β-alanine, seen in the first splitting node, with propylene glycol dividing the second branch of samples with lower β-alanine concentrations (Figure 7A). For salmon color intensity, β-alanine concentration was again the main explanatory compound (Figure 7B). For both color attributes, the higher β-alanine concentration group had less intense coloration. Glucose 1-phosphate was the second split found in the low-concentration β-alanine branch. Hypoxanthine was the primary node for aroma intensity, with higher levels corresponding to more intense aroma (Figure 7C). Higher formic acid concentrations corresponded to lower aroma intensity within the low-hypoxanthine group. In the high-formic acid subset, ADP was the final splitting variable, showing a relationship between higher concentrations and more intense aroma. Finally, a more intense root vegetable taste showed a negative relationship with alanine, and the high-alanine branch was further split by succinic acid, where lower levels related to a more intense taste (Figure 7D).

**Figure 7:**
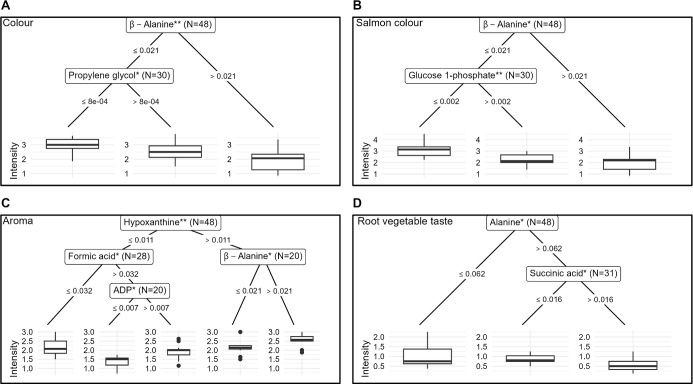
Conditional inference tree (CIT) models of all profiled metabolites as explanatory variables and each of the four key sensory attributes as dependent variables: (A) color; (B) salmon color; (C) aroma; and (D) root vegetable taste. For each attribute, nodes show splitting variables (metabolites), stars for *p*-value representation (***, *p* < 0.001; **, *p* < 0.01; *, *p* < 0.05), and subset size. Edge labels show if the next node belongs to the high or low concentration subset and at what concentration the split was found. Terminal box and whiskers plots show final subset data distribution by median, interquartile range, whiskers as 1.5 times the interquartile range, and outliers as points.

## Discussion

The objective of this work was to evaluate how protein alternatives in feed influence muscle metabolite composition and sensory properties of rainbow trout fillets. Although the sensory properties remained largely unaffected by the feed given, color, aroma, and root vegetable taste were perceived differently between products. The diets also appear to cause differences in the muscle metabolite compositions. According to the multivariate analysis, the insect-, microfungi-, and mussel-based diets were clearly separated from the control, whereas the tunicate diet was the most similar to it. It was also evident that the two insect-based diets resulted in similar metabolic profiles. Among the most influential metabolites explaining the muscle metabolome patterns, six metabolites; betaine, hypoxanthine, malonic acid, methylmalonic acid, TMAO, and 2-hydroxybutyric acid showed significant concentration differences between the diets.

### Diet Impact on Product Quality

The sensory character of the rainbow trout fillets, illustrated by the sPLS-DA model (Figure 5), showed no clear separated clustering due to diet. Hence, even though the feed metabolomes differed quite clearly, there was no striking impact on the product sensory attributes overall. The result showed that only minor sensory characteristics were altered by feed, which aligns with previous findings.^57^ However, the sPLS-DA revealed a clustering of samples from fish given the MF feed that coincided with a more pronounced root vegetable taste. A common issue with, for example, inland lake fish is an “earthy” off-flavor attributed to geosmin, a metabolite produced by freshwater cyanobacteria.^58^ One might initially think that root vegetable taste could be associated with similar “earthy” tones—but it is not to be viewed as a proxy for it—as panellists interpreted terms freely without attribute descriptions. Yet, root vegetable taste may not be typical or desirable in rainbow trout and could therefore be relevant to consumers. In this context, the findings have implications not only for MF but also for MW and CTRL, as the mixed model analysis showed no significant differences in root vegetable taste intensity between the groups. However, studies have shown that sensory education and transparent information on the benefits of a food item can enhance consumer acceptance,^59^ even when sensory attributes deviate from the typical product characteristics.^39^ This suggests that while sensory deviations exist, they may not necessarily hinder consumer acceptance if properly addressed.

As a breakdown product of adenosine triphosphate (ATP) and its four autolytic derivates (adenosine diphosphate, ADP; adenosine monophosphate, AMP; inosinic acid, IMP; and inosine), hypoxanthine accumulates in the muscle as the aerobic ATP synthesis stops, switches to anaerobic glycolysis, and the available energy eventually runs out after death.^60^ On top of this, bacterial spoilage can also facilitate the accumulation of hypoxanthine.^61^ Thus, the hypoxanthine levels can indicate the freshness of the meat. Compared to the CTRL and CIO groups, fish given MW feed had lower levels of muscular hypoxanthine.

Hypoxanthine contributes to bitter off-flavours, while its previous form (IMP) is a desired part of the umami development of cured meat products.^62^ Unless consumed soon after slaughter, a slower ATP breakdown is preferable as it provides a longer shelf life, which could be argued to favor the MW feed. Among all metabolites, hypoxanthine also appeared as the primary splitting node in the conditional inference tree analysis, where aroma intensity was set as the response variable (Figure 7C). Hypoxanthine itself is odorless, but the relation between hypoxanthine build-up and a more intensive smell supports the idea of faster degradation of the meat. MM and CTRL showed the highest estimated marginal means for aroma intensity, but this pattern for the hypoxanthine levels was only reflected in the CTRL group. Hypoxanthine is not the cause but a potential indicator of spoilage, which correlates with the development of a more pronounced aroma originating from various compounds. From a consumer perspective, a less intense aroma is generally a positive trait for fish products,^63^ which would favor the BSF-, MF-, and particularly the MW feed in this regard.

Trimethylamine-*N*-oxide (TMAO) is an osmolyte typically found in marine animals^64^ but can be found in freshwater species through exogenous sources. TMAO is formed primarily through TMA oxidation, which in turn originates directly from the diet or dietary choline, betaine, or carnitine through conversion by gut microbiota.^65^ Flavor-wise, TMAO does not contribute in itself, but through breakdown by bacteria, TMA is formed, which has the characteristic “fishy” odor that develops as the bacterial spoilage increases. TMAO was prevalent in higher concentrations in the muscle tissue of the MM diet group compared to all others, and the CIO group contained higher levels than the BSF group. Regarding the ″fishy″ odor, the less available TMAO for bacteria to convert to TMA, the better, which primarily favors BSF, but also MW, MF, and CTRL. No TMAO was found in the feed metabolomes, and the dietary precursors (betaine, choline, carnitine, and TMA) showed high concentrations for the MM feed relative to the others, which is consistent with the muscle TMAO concentrations. This pattern can also be seen in the CIO group. Based on the dietary availability of precursors, one would expect the CTRL or potentially MF to show the lowest muscular TMAO concentrations. However, when strictly looking at the mean concentrations, the BSF group has the lowest. This is surprising, as the dietary carnitine levels are among the highest for BSF. Even though the literature on dietary sources of TMAO and its subsequent expression in rainbow trout fillet is limited, studies on mice and tilapia point toward choline rather than carnitine as the major contributor to higher TMAO levels during long-term administration.^66,67^ The availability of TMAO precursors in the MM and CIO feeds could potentially explain the higher muscular levels of TMAO observed in these groups, but it cannot be said to explain why BSF muscle contains significantly lower levels than both MM and CIO. Therefore, other factors should be considered when assessing the causes of muscular TMAO concentrations, including excretion rate, kidney function, or gut microbial composition.^68^

Coloration is another key product aspect. The CIT for color intensities (Figure 7A,B) linked the low β-alanine subsets to higher overall and salmon-color intensity, with additional associations to propylene glycol and glucose 1-phosphate. However, there are no clear connections between these metabolites and the coloration of the meat. If these truly reflect coloration, it is likely an indirect signal of compounds not identified by the applied method. This is reasonable as fillet coloration can be attributed to concentrations of larger, often hydrophobic, pigment molecules like astaxanthin and canthaxanthin.^69^ Changes in rainbow trout fillet coloration have been demonstrated in several studies following the inclusion of alternative proteins in feed. Compared with fishmeal-based control diets, high levels of soy protein concentrate resulted in lighter fillets with reduced redness,^70^ and decreased pigmentation have been reported in fish given blue mussel diets.^28^ As the control used in this study was designed to reflect a relevant commercial diet rather than a pure fishmeal one, parallels to these studies are tough to make. Nevertheless, it is worth noting that MW and CIO had a lower overall coloration than the CTRL group, and that BSF stands out as more intense than these two in salmon meat color. The difference between the two insect diets is a noteworthy contrast to earlier reported findings, where black soldier fly and mealworm diets both produced fillets with coloration similar to those of fishmeal-fed rainbow trout.^71,72^ As more intense salmon colored fillets has been associated with greater consumer preference,^73^ feeds that promote such pigmentation—such as BSF in this case—may, therefore, be considered more desirable.

Taking the observations together, diet-related differences in metabolite concentrations among fillets may have sensory implications, though links between sensory attributes and metabolites were unclear. MW, BSF, and MF feeds resulted in comparatively lower muscular hypoxanthine and TMAO concentrations, which could signal slower deterioration and a less dramatic “fishy” odor development. BSF- and MF-fed fish did not have a particularly intense aroma, and MW-fed fish had the lowest estimated marginal mean. These diets may thus contribute to a more consumer-friendly odor, though MF stands out taste-wise given the evident root vegetable tones. On top of this, BSF had a more intense coloration, which is also favorable in terms of consumer preference. MM, CIO, and CTRL diets can be argued to have slightly more limitations, including higher hypoxanthine levels (CIO, CTRL), elevated TMAO concentrations (MM and, potentially, CIO), more intense aroma (MM, CTRL), and an evident root vegetable taste (MF).

In the context of sensory characteristics and biomarkers, insect-based feeds appear to offer possible advantages if taken together. Between these, MW seems slightly more favorable regarding freshness and sensory neutrality, while BSF shows strengths in fillet color and TMAO reduction. Nonetheless, the perceived differences by the sensory panel showed that the alternative protein sources resulted in an overall mild impact on the sensory properties of the rainbow trout fillets. Therefore, all tested feeds can be considered viable options from a consumer perspective. These results can be considered exploratory, and future studies would be needed to assess shelf life altering aspects of diets, as well as targeted studies on the highlighted metabolites and metabolic effects discussed.

### Dietary Influences on Metabolism

In the sPLS-DA analysis for muscle metabolites, clustering indicates that the alternative feeds contribute to changes in the metabolome composition. While the muscle metabolomes of insect- and microfungi-based feeding groups overlap, they cluster apart from the control. The marine feeds slightly overlay the three previously mentioned diet groups and the control, but they are unique in clustering and spread. The CIO group overlaps with the control group to a large extent, and MM takes on the more unique spread of the two. It is reasonable to expect a more evident difference, given that the CIO-feed would have had no inclusion of mussel meal. Even though they share living environment and feeding strategy, tunicates are vastly different from blue mussels, both morphologically and phylogenetically.^32,74^

Betaine serves several functions for aquatic animals, including as an osmolyte to support cell integrity under environmental stress,^75^ regulation of lipid oxidation and deposition,^76^ and as a key precursor for methionine and *S*-adenosylmethionine (SAM), essential for RNA, DNA, and protein synthesis.^77^ Betaine originates directly from dietary sources or through oxidation of choline and can, to an extent, reduce the need for dietary choline and methionine.^78^ In this study, BSF and MW diets resulted in higher muscle concentrations of betaine compared to the CTRL and MF groups, with similar patterns observed in the feed betaine content (Figure 2). However, while the MM feed contained the highest levels of betaine and choline, the group’s muscular concentrations were only higher than the CTRL, potentially due to the feed’s low methionine content. The possible explanation is that, for the MM diet, more betaine and choline may have been redirected toward methionine production, limiting their availability for muscle incorporation.

Betaine and TMAO share functions as osmolytes, and while TMAO is naturally more abundant in the inhabitants of marine environments, betaine can be found in a wide variety of aquatic and terrestrial organisms.^79^ While both metabolites serve beneficial physiological purposes in fish, they show opposing effects on human health. Higher levels of plasma TMAO can be linked to, especially, cardiovascular diseases,^80^ while betaine shows several positive health effects.^65^ In the case of farmed freshwater rainbow trout, where the TMAO is introduced through diet, low-TMAO diets could be a better choice.

Malonic acid concentrations were higher in the skeletal muscle tissue of the MM group compared to all other groups except CIO. This aligns with previous studies on arctic charr, where mussel-based diets were also associated with elevated muscular malonic acid levels.^47^ Malonic acid is primarily known as a competitive inhibitor of succinate dehydrogenase, affecting the TCA cycle and the electron transport chain, impacting energy status.^81,82^

The muscular concentration of methylmalonic acid was found in lower levels in the CTRL group compared to MF and MM. When vitamin B_12_ levels are insufficient for the production of succinyl-CoA from the CoA-linked form of methylmalonic acid (methylmalonyl-CoA), an accumulation of methylmalonic acid follows, which is why the compound can be used as a biomarker for B_12_ deficiency.^83^ Replacement of fish meal and fish oil with plant-based alternatives has been shown to reduce available dietary B_12_, and the expected plant-derived increase of methylmalonic acid has been confirmed for gilthead sea bream (*Sparus aurata*) serum.^84^ However, Hansen et al.^85^ reported that the muscular content of B_12_ for cod (*Gadus morhua*) was constant even at 100% replacement of fish protein.

Both malonic acid and methylmalonic acid are linked to the TCA cycle and energy metabolism, which is also true for 2-hydroxybutyric acid. Although not directly involved in the TCA cycle, 2-hydroxybutyric acid is created as a byproduct during the production of 2-ketobutyric acid from amino acid catabolism and glutathione anabolism. 2-Ketobutyric acid then feeds into the TCA cycle as succinyl-CoA after conversion through propionyl-CoA;^86^ hence, measured levels could be linked to energy metabolism. 2-Hydroxybutyric acid concentrations have also been shown to increase under hypoxia or anaerobic glycolysis.^87^ These are conditions under which hypoxanthine would be expected to accumulate faster, which, to an extent, is reflected in the results. Muscular concentrations in CIO show significantly higher levels than MW of hypoxanthine and 2-hydroxybutyric acid, and the overall patterns follow this for the other groups. However, the patterns do not align perfectly and are not statistically supported, so no conclusions can be made regarding the connections between hypoxanthine and 2-hydroxybutyric acid.

While the present study provides findings regarding the effect of alternative feeds on muscle metabolism and sensory properties of rainbow trout fillets, some limitations must be acknowledged. The first is the use of two replicate tanks in the study. However, tank effects have been taken into account during statistical analyses, which could be performed on an individual level as all fish were tagged and fully traceable throughout the study. Another limitation is in the detectable range of metabolites. Though potentially relevant to sensory aspects and metabolism, lipids were excluded. The rationale is that the emphasis was on replacing feed protein, rather than the oil fraction. Furthermore, in comparison to alternatives such as liquid chromatography–mass spectrometry (LC–MS), NMR is limited in the number of metabolites it can detect, particularly low-abundance compounds. However, the advantage of NMR is that it is highly reproducible and provides reliable quantitative information for detected metabolites.

In summary, the replacement of soy protein with black soldier fly, mealworm, microfungi, and blue mussel sources in rainbow trout feeds appears to impact the metabolome of white skeletal muscles. The observed muscular concentrations of key metabolites within the groups suggest potential influences on cellular processes such as osmoregulation (betaine and TMAO), energy metabolism (hypoxanthine, malonic acid, methylmalonic acid, and 2-hydroxybutyric acid), and amino acid catabolism (2-hydroxybutyric acid and betaine). From a product perspective, all diets retained similar core sensory characteristics, with only minor deviations such as variations in root vegetable taste and color intensity. These findings indicate that while these alternative proteins can alter muscle metabolism of rainbow trout, their influence on consumer-relevant sensory traits is limited, suggesting that the feeds are viable for maintaining fillet quality.

## Supplementary Material



## Notes

Additional details of the data analysis, including representative ^1^H NMR spectra for feed and muscle tissue polar extracts, full list of sensory attributes, and statistical outputs (PDF)
