# Supplementary material for: Broadly Sourced Alternative Proteins Alter Muscle Metabolome While Maintaining Sensory Quality in Rainbow Trout (Oncorhynchus mykiss)
Source: J Agric Food Chem. 2025 Oct 22;73(44):28511–23. doi: 10.1021/acs.jafc.5c07909 (PMC12593341; doi:10.1021/acs.jafc.5c07909)
Supplement: Supplementary file 1 [file jf5c07909_si_001.pdf]

Supporting Information

Broadly Sourced Alternative Proteins Alter Muscle Metabolome While Maintaining Sensory Quality in Rainbow Trout (*Oncorhynchus mykiss*)

Pontus Gunnarsson[a]*, Hanna Eriksson Röhnisch[b], Mihaela Mihnea[c], Aleksandar Vidakovic[a], Markus Langeland[d], Anders Kiessling[a], and Johan Dicksved[a]

[a]*Department of Applied Animal Science and Welfare, Swedish University of Agricultural Sciences, 756 51 Uppsala, Sweden*

[b]*Department of Molecular Sciences, Swedish University of Agricultural Sciences, 750 07 Uppsala, Sweden*

[c]*Sense Lab, School of Hospitality, Culinary Arts and Meal Science, Örebro University, 701 12 Örebro, Sweden*

[d]*Department of Food Research and Innovation, RISE Research Institutes of Sweden, 402 29 Göteborg, Sweden*

*Corresponding author. Email: pontus.gunnarsson@slu.se

Table of content:

Table S1: Complete sensory attribute list

Table S2: Identified metabolites in feed and white skeletal muscle tissue.

Figure S1: ^{1}H-NMR representative spectrum of feed sample polar extracts.

Figure S2: ^{1}H-NMR representative spectrum of white muscle tissue sample polar extracts.

Figure S3: Multivariate observation outlier detection (PCA) for feed samples.

Figure S4: Multivariate observation outlier detection (PCA) for muscle samples.

Figure S5: Multivariate observation outlier detection (PCA) for sensory scores.

Figure S6: Effect of normalisation on feed metabolite concentrations.

Figure S7: Effect of normalisation on muscle metabolite concentrations.

Figure S8: Effect of normalisation on sensory attribute scores.

Table S3: Model comparisons for individual muscle metabolites.

Table S4: Mixed effects model comparisons for sensory attributes.

Table S1: List of attributes used for sensory analysis of rainbow trout fillets. Attributes were presented to panellists in Swedish and translated afterwards.

Attribute	Original attribute description (Swedish)	Category
Colour intensity	Färgintensitet	Appearence
Salmon colour intensity	Laxfärgintensitet	Appearence
Aroma intensity	Doftintensitet	Aroma
Fresh fish aroma intensity	Färsk fisk - Doftintensitet	Aroma
Cured fish aroma intensity	Lagrad fisk - Doftintensitet	Aroma
Oceanic aroma intensity	Hav - Doftintensitet	Aroma
Foul aroma intensity	Unkenhet - Doftintensitet	Aroma
Mineral aroma intensity	Mineral - Doftintensitet	Aroma
Oil-like aroma intensity	Oljeliknande - Doftintensitet	Aroma
Root vegetable aroma intensity	Rotfrukt - Doftintensitet	Aroma
Shellfish aroma intensity	Skaldjur - Doftintensitet	Aroma
Sweet taste intensity	Sötma - Smakintensitet	Taste
Sour taste intensity	Syra - Smakintensitet	Taste
Salty taste intensity	Sälta - Smakintensitet	Taste
Bitter taste intensity	Beska - Smakintensitet	Taste
Umami taste intensity	Umami - Smakintensitet	Taste
Metal taste intensity	Metal - Smakintensitet	Taste
Fresh fish taste intensity	Färsk fisk - Smakintensitet	Taste
Cured fish taste intensity	Lagrad fisk - Smakintensitet	Taste
Grassiness taste intensity	Gräsighet - Smakintensitet	Taste
Foul taste intensity	Unkenhet - Smakintensitet	Taste
Root vegetable taste intensity	Rotfrukt - Smakintensitet	Taste
Shellfish taste intensity	Skaldjur - Smakintensitet	Taste
Chewiness	Tuggmotstånd	Texture

Table S2: Identified metabolites in feed (F) and white skeletal muscle tissue (M) polar fraction extracts. Multiplicity is defined as: s: singlet; d: doublet; t: triplet; q: quartet; m: multiplet; dd: doublet of doublets; ddd: doublet of doublets of dublets; dt: doublet of triplets. Signals within the water region (4.5–**5.1 ppm) were excluded. Reporter signals are marked in bold and specified by “[f]” if unique to feed spectra, or “[m]” for muscle.**

Metabolite	Library signals; δ/ppm (multiplicity)
1. 2-Aminobutyric acid (FM)	3.71 (t), 1.90 (m), **0.97** (t)
2. 2-Hydroxybutyric acid (M)	3.99 (dd), 1.73 (m), 1.64 (m), **0.89** (t)
3. 3-Aminoisobutanoic acid (M)	3.1 (dd), 3.03 (dd), 2.59 (m), **1.18** (d)
4. 4-hydroxybenzoate (F)	**7.79** (d), 6.91 (d)
5. 4-Pyridoxic acid (F)	7.84 (s), **2.44** (s)
6. Acetic acid (M)	**1.91** (s)
7. Acetylcarnitine (M)	5.60 (q), 3.84 (dd), 3.59 (d), 3.17 (s), 2.63 (dd), 2.50 (dd), **2.13** (s)
8. Adenosine (F)	8.34 (s), 8.25 (s), **6.06** (d), 4.42 (dd), 4.30 (m), 3.91 (dd), 3.83 (dd)
9. ADP (M)	**8.50** (s), 8.26 (s), 6.13 (d), 4.36 (m), 4.21–4.24 (m)
10. Alanine (FM)	3.78 (q), **1.47** (d)
11. AMP (FM)	**8.59** (s), 8.26 (s), 6.13 (d), 4.36 (td), 4.01 (ddd), 3.99 (ddd)
12. Anserine (M)	8.28 (d), 8.20 (s), 7.07 (s), 3.76 (s), 3.19–3.22 (m), 3.02 (dd), **2.65–2.70** (m)
13. Aspartic acid (F)	3.89 (dd), **2.80** (dd), 2.67 (dd)
14. beta-Alanine (FM)	3.17 (t), **2.55** (t)
15. Betaine (FM)	**3.89** (s), 3.25 (s)
16. Carnitine (F)	3.40–3.43 (m), 3.21 (s), **2.45** (dd), 2.41 (dd)
17. Cholic acid (F)	**0.70** (s; 0.70–4.07, 30 peak clusters)
18. Choline (FM)	4.06 (m), 3.51 (m), **3.19** (s)
19. Creatine (FM)	3.92 (s), **3.02** (s)
20. Creatinine (FM)	**4.05** (s), 3.03 (s)
21. Cytosine (F)	7.50 (d), **5.97** (d)
22. Deoxyuridine (F)	**7.84** (d), 6.27 (t), 5.88 (d), 4.04 (m), 3.83 (dd), 3.76 (dd), 2.40–2.41 (m)
23. Dihydrothymine (M)	3.49 (dd), 3.17 (dd), 2.78 (m), **1.19** (d)
24. Dimethylamine (FM)	**2.71** (s)
25. Dimethylglycine (M)	3.71 (s), **2.92** (s)
26. Formic acid (FM)	**8.44** (s)
27. Fumaric acid (FM)	**6.51** (s)
28. gamma-Aminobutyric acid (F)	3.00 (t), **2.29** (t), 1.89 (m)
29. Glucose 1-phosphate (M)	**5.45** (dd), 3.91 (dt), 3.87 (q), 3.77 (t), 3.74 (dd)
30. Glutamic acid (FM)	3.75 (dd), **2.36** (m), 2.12 (m), 2.04 (m)
31. Glycerophosphocholine (FM)	3.61 (dd), 3.94 (m), 3.90 (m), 3.87 (m), 3.67 (dd), 3.67 (m), 3.61 (dd), **3.22** (s)
32. Glycine (FM)	**3.55** (s)
33. Guanosine (F)	**7.99** (s), 5.90 (d), 4.40 (dd), 4.22 (q), 3.88 (dd), 3.81 (dd)
34. Histamine (M)	**7.93** (s), 7.07 (s), 3.27 (t), 2.97 (t)
35. Hypoxanthine (FM)	**8.20** (s), 8.18 (s)
36. Inosine (FM)	8.34 (s), 8.23 (s), **6.09** (d), 4.43 (dd), 4.27 (q), 3.90 (dd), 3.83 (dd)
37. Inosinic acid (FM)	**8.56** (s), 8.22 (s), 6.13 (d), 4.36 (m), 4.03 (m), 4.00 (m)
38. Isoleucine (FM)	3.67 (d), 1.97 (m), 1.46 (m), 1.25 (m), **1.00** (d), 0.93 (t)
39. Lactic acid (FM)	4.11 (q), **1.32** (d)

Table S2: (Continued)

**Metabolite**	**Library signals; δ/ppm (multiplicity)**
40. Leucine (FM)	3.73 (m), 1.73 (m), 1.70 (m), 1.67 (m), **0.95** (d), 0.94 (d)
41. Lysine (M)	3.75 (t), **3.02**^{f} (t), 1.92 (m), 1.89 (m), **1.72**^{m} (tt), 1.50 (m), 1.43 (m)
42. Malonic acid (FM)	**3.12** (s)
43. Methionine (FM)	3.85 (dd), 2.63 (t), 2.18 (m), **2.13** (s), 2.11 (m)
44. Methylmalonic acid (FM)	3.17 (q), **1.21** (d)
45. myo-Inositol (F)	4.06 (t), **3.61** (t), 3.53 (dd), 3.27 (t)
46. N-Acetyl-L-aspartic acid (F)	7.92 (d), 4.38 (m), 2.68 (dd), 2.48 (dd), **2.01** (s)
47. Niacinamide (FM)	8.93 (dd), **8.70** (dd), 8.24 (ddd), 7.59 (dd)
48. Nicotinic acid (F)	8.93 (dd), **8.60** (dd), 8.25 (ddd), 7.52 (dd)
49. Oxypurinol (F)	**8.19** (s)
50. Pantothenic acid (FM)	8.00 (s), 3.98 (s), 3.51 (d), 3.43 (q), 3.39 (d), 2.41 (t), **0.92** (s), 0.88 (s)
51. Phenylalanine (FM)	**7.42** (m), 7.36 (m), 7.32 (m), 3.99 (dd), 3.28 (dd), 3.12 (dd)
52. Phosphorylcholine (F)	4.16 (m), 3.58 (t), **3.21** (s)
53. Proline (FM)	4.12 (dd), 3.41 (m), **3.33** (m), 2.34 (m), 2.06 (m), 2.02 (m), 1.97 (m)
54. Propylene glycol (FM)	3.87 (m), 3.54 (dd), 3.44 (dd), **1.13** (d)
55. Pyroglutamic acid (F)	4.17 (dd), **2.5** (m), 2.41 (dd), 2.38 (dd), 2.02 (m)
56. Pyruvic acid (M)	**2.36** (s)
57. Sarcosine (M)	**3.60**^{f} (s), **2.74**^{m} (s)
58. Succinic acid (FM)	**2.40** (s)
59. Sucrose (F)	5.40 (d), **4.21** (d), 4.04 (t), 3.88 (m), 3.80–3.83 (m), 3.76 (t), 3.68 (t), 3.55 (dd), 3.47 (t)
60. Taurine (FM)	**3.42** (t), 3.25 (t)
61. Threonine (FM)	4.25 (m), **3.58** (d), 1.32 (d)
62. Thymidine (F)	**7.63** (s), 6.29 (t), 4.01 (dt), 3.84 (dd), 3.76 (dd), 2.37 (dd), 1.89 (s)
63. Thymine (F)	7.36 (s), **1.86** (s)
64. TMA (FM)	**2.89** (s)
65. TMAO (M)	**3.26** (s)
66. Trehalose (F)	**5.18** (d), 3.85–3.86 (m), 3.81 (m), 3.76 (m), 3.64 (dd), 3.45 (t)
67. Trigonelline (F)	**9.12** (s), 8.83 (d), 8.83 (d), 8.07 (t), 4.43 (s)
68. Tryptophan (F)	**7.72** (d), 7.53 (d), 7.31 (s), 7.27 (t), 7.19 (t), 4.05 (q), 3.48 (dd), 3.30 (dd)
69. Tyramine (F)	**7.21** (d), 6.89 (d), 3.23 (t), 2.92 (t)
70. Tyrosine (FM)	**7.18** (ddd), 6.89 (ddd), 3.93 (t), 3.19 (dd), 3.03 (dd)
71. UMP (F)	8.09 (d), **5.98** (dd), 4.41 (t), 4.34 (t), 4.25 (m), 3.96–4.02 (m)
72. Uracil (FM)	7.53 (d), **5.79** (d)
73. Uridine (F)	7.86 (d), **5.90** (dd), 4.34 (t), 4.22 (t), 4.12 (ddd), 3.90 (dd), 3.80 (dd)
74. Valine (FM)	3.60 (d), 2.26 (m), **1.03** (d), 0.98 (d)

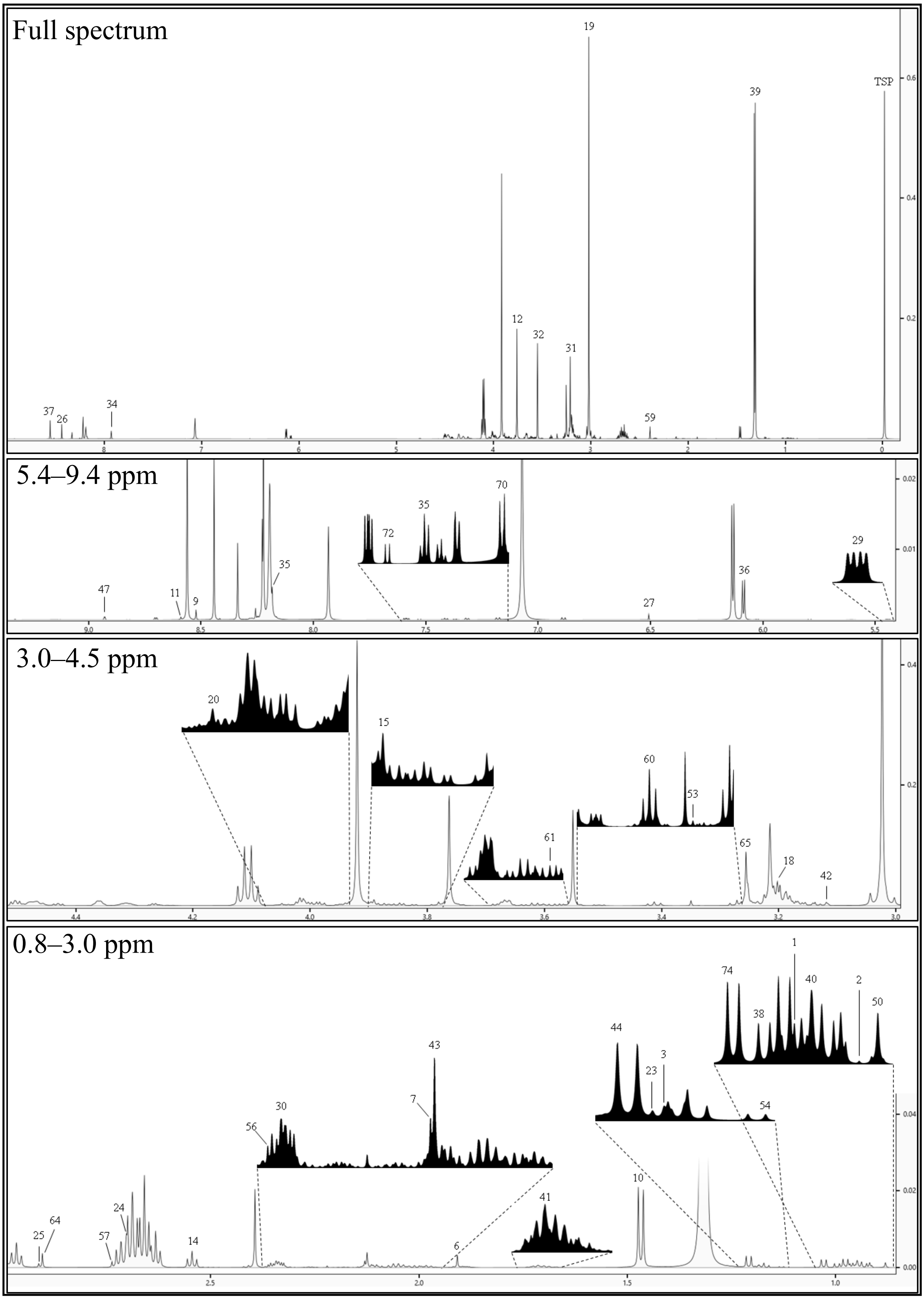

Figure S1: Representative ^{1}H-NMR spectrum of muscle polar extract spliced into segments to increase visibility. Annotated numbers mark the chosen signal peak for the corresponding metabolite in Table S2.

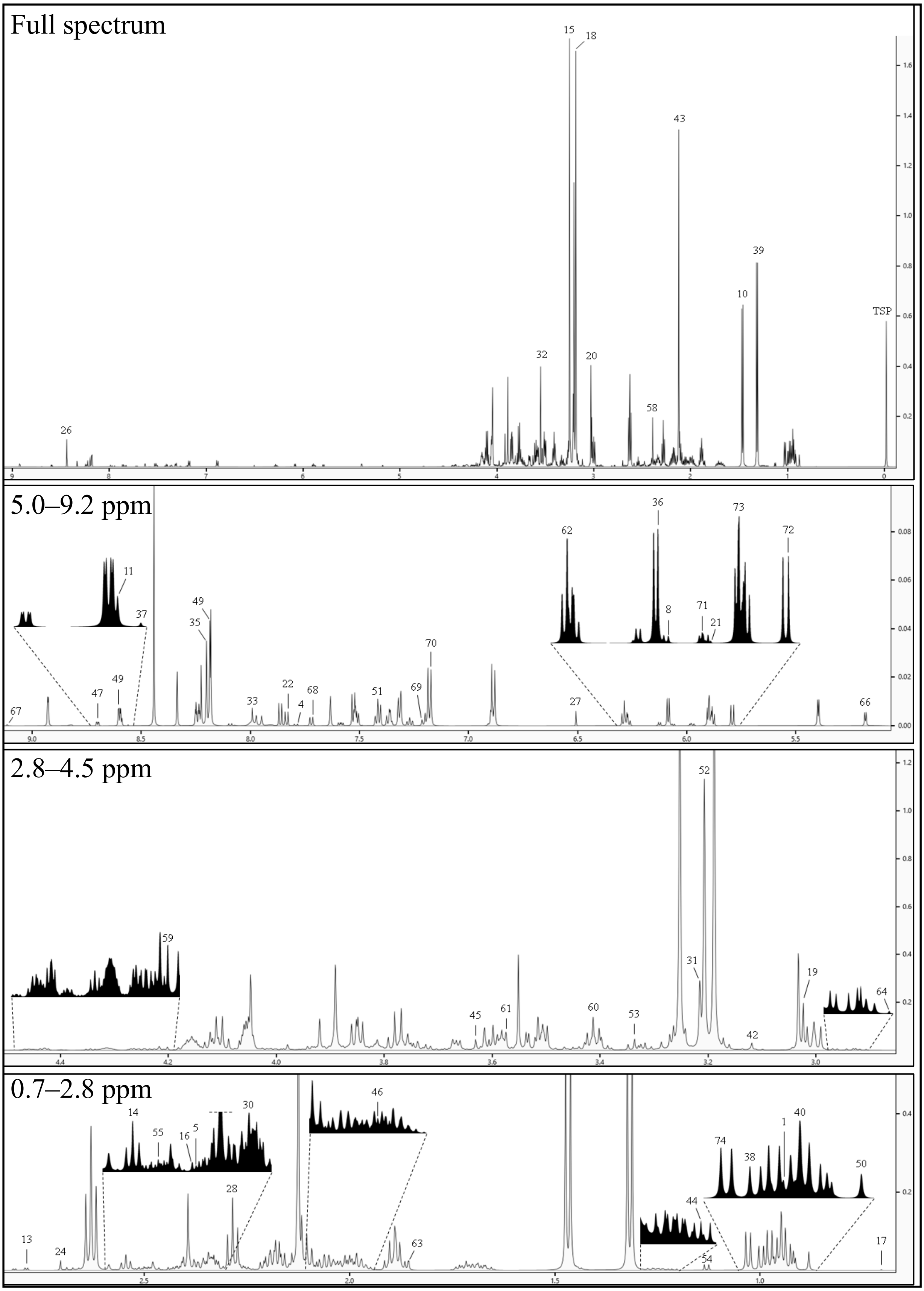

Figure S2: Representative ^{1}H-NMR spectrum of muscle polar extract spliced into segments to increase visibility. Annotated numbers mark the chosen signal peak for the corresponding metabolite in Table S2.

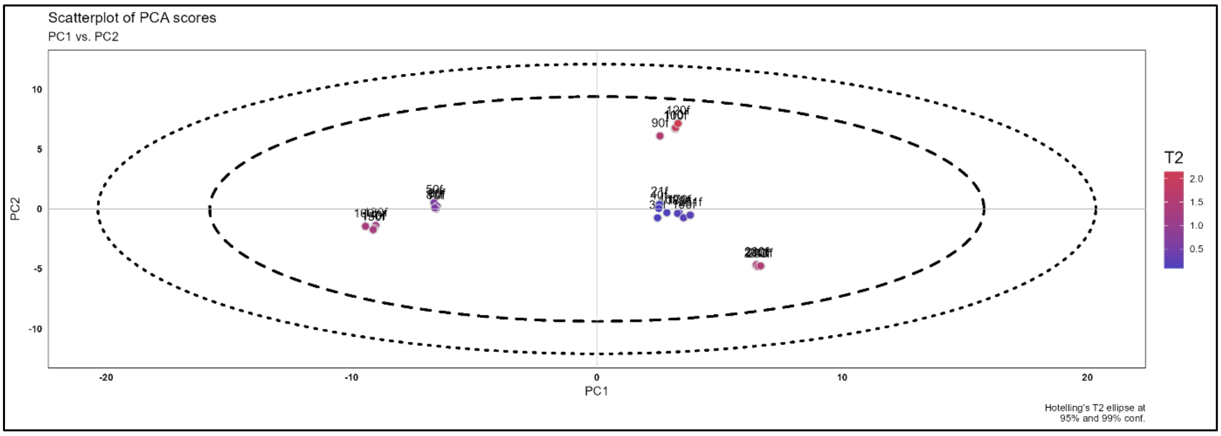

Figure S3: PCA with Hotelling's T^2 for observation outlier detection of feed samples. Points outside the inner circle (95% confidence limit) are considered outliers.

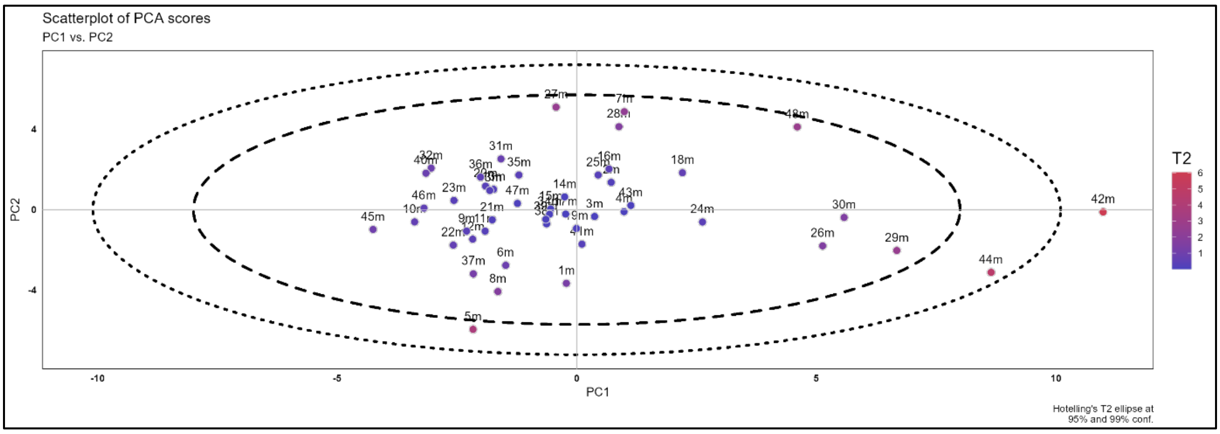

Figure S4: PCA with Hotelling's T^2 for observation outlier detection of feed samples. Points outside the inner circle (95% confidence limit) are considered outliers.

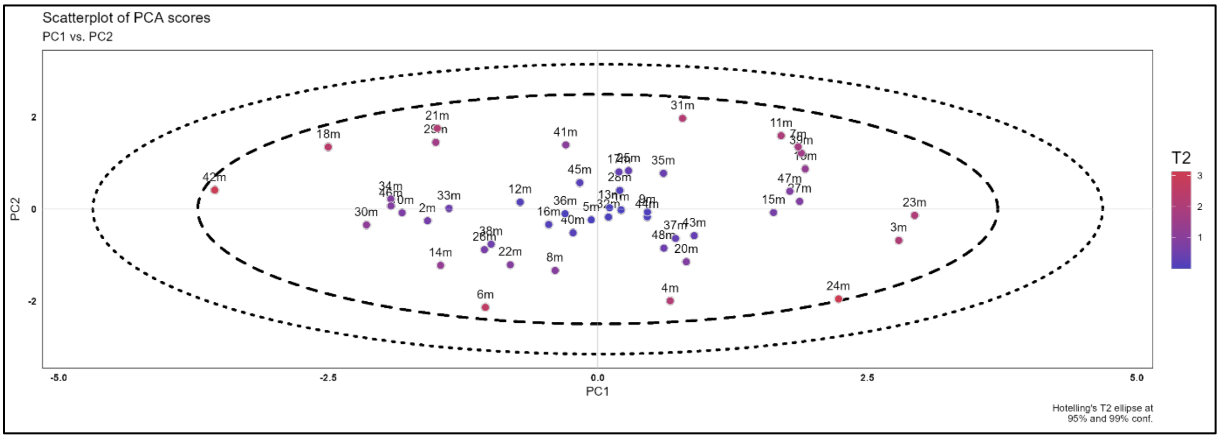

Figure S5: PCA with Hotelling's T^2 for observation outlier detection of sensory scores. Points outside the inner circle (95% confidence limit) are considered outliers.

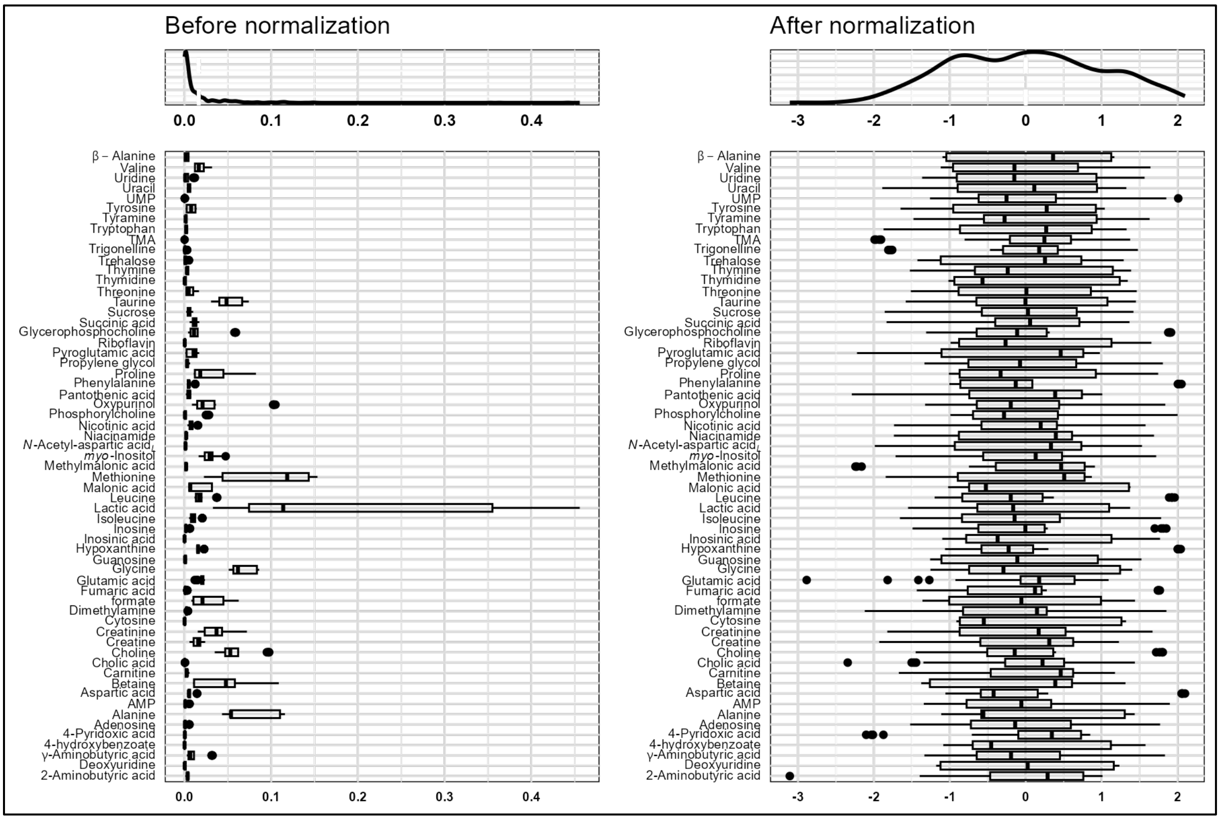

Figure S6: Feed polar extract metabolite concentrations before and after normalisation by sum, log-10 transformation, and auto-scaling.

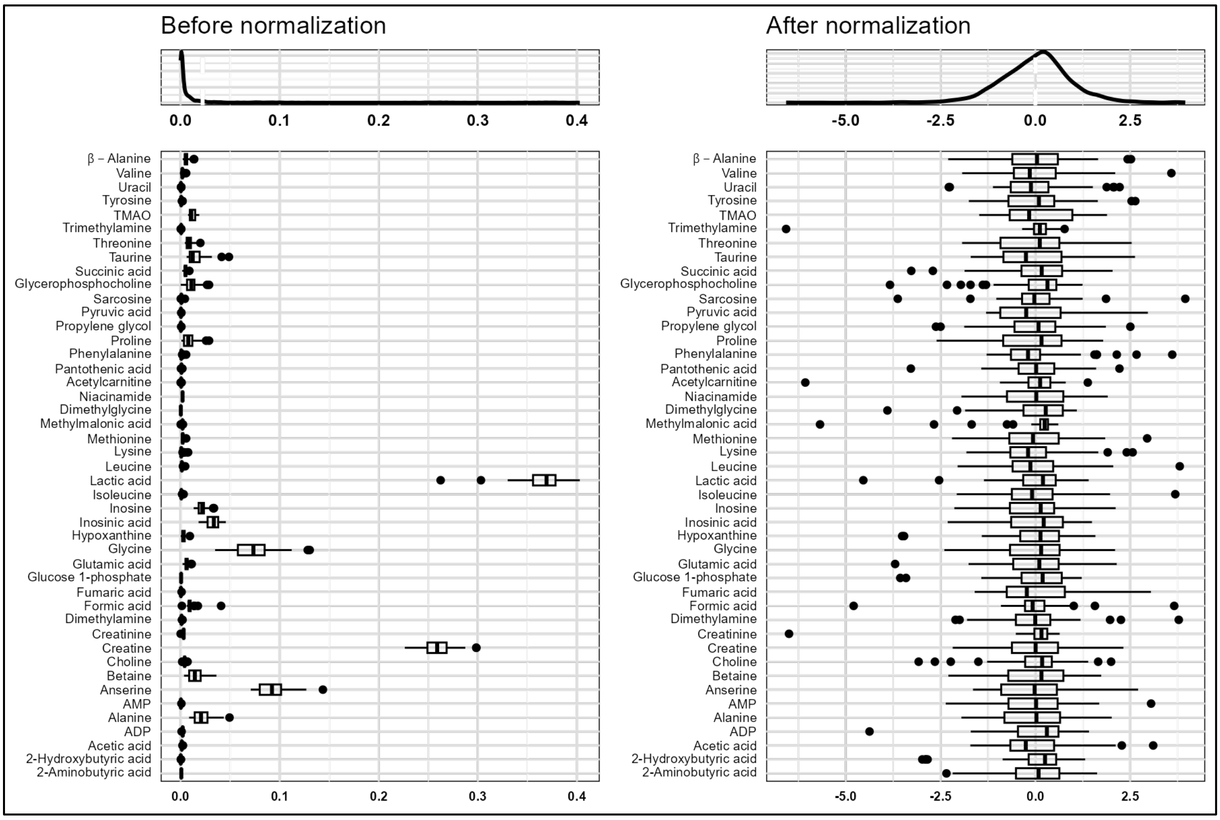

Figure S7: Muscle polar extract metabolite concentrations before and after normalisation by sum, log-10 transformation, and auto-scaling.

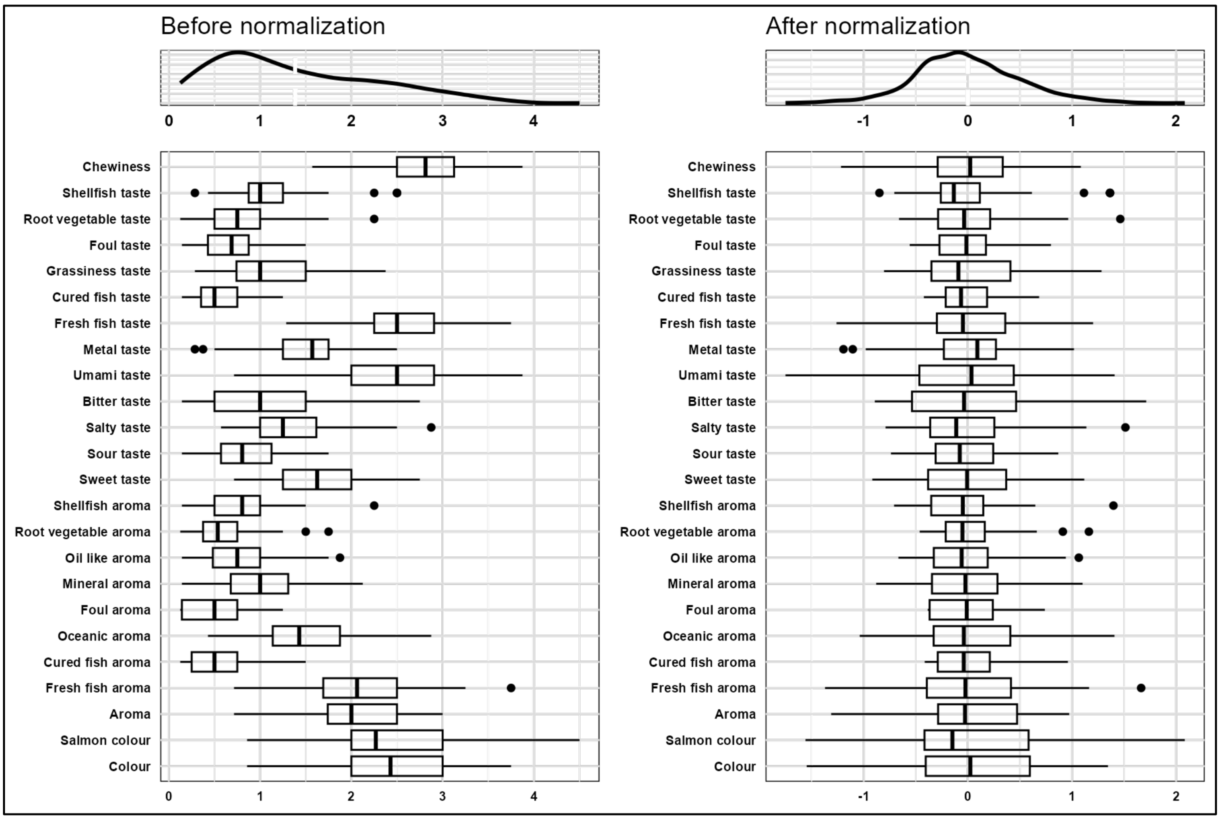

Figure S8: Sensory scores (Rate-All-That-Apply) data distribution before and after normalisation by sum, and mean-scaling.

Table S3: Model comparisons for selection of individual muscle metabolites with groupwise concentration differences. The fixed-effects model was designed as "Metabolite ~ Feed" and the nested effects model as "Metabolite ~ Feed / Tank".

Metabolite	FDR-adjusted p-value (Fixed-effects model)	FDR-adjusted p-value (Nested effects model)	Main effect
Hypoxanthine	0.0292*	0.5456 (n.s.)	Diet
Methylmalonic acid	0.0292*	0.0372*	Diet
2-Hydroxybutyric acid	0.0067**	0.0841 (n.s.)	Diet
Betaine	0.0014**	0.1697 (n.s.)	Diet
Malonic acid	<0.001***	0.6243 (n.s.)	Diet
TMAO	<0.001***	0.0457*	Diet
Valine	0.0552 (n.s.)	0.4337 (n.s.)	Diet
Dihydrothymine	0.0424*	0.019*	Tank
Lactic acid	0.0309*	0.019*	Tank
Creatinine	0.1208 (n.s.)	0.019*	Tank
Ethanol	0.7084 (n.s.)	0.0274*	Tank
Propylene glycol	0.3142 (n.s.)	0.0132*	Tank

Table S4: Mixed effect model comparisons of sensory attribute scores. The full model includes diet as independent variable (Sensory attribute ~ Feed * (1|Judge)) in contrast to the reduced model (Sensory attribute ~ Feed * (1|Judge)). Models were fitted using maximum likelihood (ML).

Sensory attribute	AIC (full model)	AIC (reduced model)	p-value
Colour intensity	961.38	944.64	<0.001***
Salmon colour intensity	981.52	971.00	<0.001***
Aroma intensity	1012.51	1005.76	0.0050**
Fresh fish aroma	1087.69	1086.90	0.0555 (n.s.)
Cured fish aroma	728.44	735.20	0.6626 (n.s.)
Oceanic aroma	1052.69	1060.22	0.7802 (n.s.)
Foul aroma	590.51	593.76	0.2398 (n.s.)
Mineral aroma	978.45	986.26	0.8227 (n.s.)
Oil-like aroma	888.96	894.10	0.4329 (n.s.)
Root vegetable aroma	824.50	829.60	0.4274 (n.s.)
Shellfish aroma	878.07	881.63	0.2655 (n.s.)
Sweet taste	972.87	979.35	0.6204 (n.s.)
Sour taste	905.08	911.30	0.5819 (n.s.)
Salty taste	1018.44	1020.73	0.173 (n.s.)
Bitter taste	922.83	927.77	0.4083 (n.s.)
Umami taste	1090.98	1099.49	0.9144 (n.s.)
Metal taste	1118.90	1123.66	0.3866 (n.s.)
Fresh fish taste	1210.41	1214.38	0.3028 (n.s.)
Cured fish taste	748.75	755.35	0.638 (n.s.)
Grassiness taste	1052.83	1057.62	0.3903 (n.s.)
Foul taste	829.47	837.72	0.8823 (n.s.)
Root vegetable taste	982.34	964.26	<0.001***
Shellfish taste	960.02	967.02	0.7003 (n.s.)
Chewiness	993.19	994.21	0.1098 (n.s.)
